# Does market access drive trade growth? Evidence from China

**DOI:** 10.1371/journal.pone.0334661

**Published:** 2025-10-23

**Authors:** Jianxin Mao, Chaoran Pan, Luoxin Wang, Ming Liu

**Affiliations:** 1 Department of Management Science, Yunnan University, Kunming, China; 2 School of Emergency Management science and Engineering, University of Chinese Academy of Sciences, Beijing, China; Gabriele d'Annunzio University of Chieti and Pescara: Universita degli Studi Gabriele d'Annunzio Chieti Pescara, ITALY

## Abstract

Against the backdrop of a turbulent global trade environment, high-quality development of foreign trade is an important driving force for the recovery and sustainable development of both China and other economies worldwide. This study investigates the influence of market access on the high-quality development of foreign trade, using panel data from 260 Chinese cities from 2014 to 2023. A fixed-effects model explores the nonlinear relationship, the transmission mechanisms of industrial structure advancement and economic agglomeration, and the moderating effect of industrial rationalization. The results reveal a significant positive U-shaped relationship: at low levels, market access improvements suppress trade quality, but beyond a critical threshold, the effect turns strongly positive. Mechanism analysis confirms that industrial structure advancement and economic agglomeration are key pathways through which market access promotes trade quality. Moreover, industrial rationalization exerts a moderating effect that reinforces the impact of market access. These findings enrich the understanding of foreign trade upgrading spatial drivers and highlight the nonlinear dynamics of market access in shaping trade quality.

## 1. Introduction

The current global foreign trade landscape is fraught with challenges. On the one hand, long-standing domestic market segmentation in various countries hampers the flow of trade between markets, increases the cost of production inputs, and impedes the advancement of production activities along the global value chain [1]. On the other hand, insufficient global economic momentum, coupled with the rise of unilateralism and protectionism, further constrains foreign trade development. As one of the founders of development economics, the German historical school economist Friedrich List pointed out in his seminal work The National System of Political Economy (1841) that dismantling internal market segmentation to promote market connectivity, as well as leveraging global markets to develop export trade vigorously, are two essential pathways for late-developing countries to catch up with advanced economies. High-quality trade development is a multi-dimensional and dynamically evolving concept in increasingly complex and volatile global markets. Its core lies in transcending the traditional model focused solely on scale expansion and shifting towards a comprehensive competitiveness upgrade centered on innovation-driven growth, structural optimization, efficiency enhancement, robust resilience, and sustainability [2–4]. This concept applies to China and represents a shared direction for transforming the global trading system. Seizing strategic opportunities, breaking down market fragmentation, innovating in cross-border factor integration, and enhancing supply chain resilience have become critical imperatives for fully unleashing comparative advantages in foreign trade and promoting the synergistic development of domestic and international markets.

Market access refers to a city’s ability to conveniently exchange economic factors such as goods, services, and information with other regions, reflecting its accessibility to other markets in the economic space network [5]. Since Harris (1954) first introduced the concept of Market Access [6], scholars have widely employed it to measure the market distance between cities and the scale of destination markets, thereby capturing the scope of accessible external potential markets. First, market access fundamentally depends on improving transportation infrastructure [7]. Enhanced transportation accessibility can foster cross-regional market integration [8]. Although the influence of transportation infrastructure on regional market integration exhibited a gradual decline between 2010 and 2019 [9], on the whole, it still improves foreign trade conditions, lowers the costs of import and export activities for enterprises, and generates both “international gateway direct access effects” and “domestic transportation advantage effects,” thereby facilitating the construction of an integrated national market [10]. Second, market access accelerates the circulation of goods and factors, enhancing the efficiency of resource allocation, which is vital for market integration and smooth trade cycles and is thus key to achieving high-quality economic development [1]. Improvements in market access reflect strengthened inter-city connectivity and reduced geographical distance and time costs between regions, which not only enhance the efficiency of factor mobility but also optimize the allocation of resources, thereby catalyzing the restructuring and upgrading of supply chains, industrial chains, and trade networks [11], providing endogenous momentum for trade upgrading.

In this sense, improving market access reduces trade costs and institutional barriers, enhances supply chain resilience, and facilitates the transformation of economic agents from market access advantages to rule-setting advantages [12], thereby promoting the qualitative and efficiency-driven upgrading of foreign trade. However, the relationship between market access and foreign trade development will likely involve more complex and indirect mechanisms. Several studies have found that improvements in market access driven by infrastructure expansion exhibit a significant U-shaped relationship with market segmentation [13]. Although eliminating market segmentation is an inherent requirement for high-level opening-up and foreign trade optimization, existing research suggests that market segmentation may facilitate firms’ entry into international markets, enabling economies of scale, increasing export shares, and enhancing export willingness [1].

Thus, the impact of market access on the high-quality development of foreign trade entails a complex and as-yet-unresolved set of mechanisms.

Building on the existing literature, this study designs an empirical framework to explore the mechanisms through which market access influences the high-quality development of foreign trade. The main contributions of this study are as follows: (1) This study reveals the non-linear mechanism through which market access affects the high-quality development of foreign trade, overcoming the limitations of traditional linear analyses and offering a new perspective for understanding the upgrading paths of cities at different development stages. (2) It constructs a multi-layered analytical framework that identifies the dual mediation effects of industrial structure advancement and economic agglomeration, while also uncovering the moderating role of industrial rationalization, thereby deepening the understanding of the internal mechanisms linking market access to trade quality. (3) Improving methodology. This study employs electronic maps with higher precision and richer data to calculate market access indicators, overcoming the accuracy limitations of traditional measurements. Dual machine learning is introduced for model validation, mitigating model selection bias inherent in conventional approaches. (4) By employing city-level evidence from China, this study fills the gap in existing literature that mainly focuses on firms or provinces. It provides meso-level evidence for development economics and new economic geography, and offers valuable policy implications for regions with similar development contexts.

## 2. Research background and theoretical analysis

Compared to transportation infrastructure alone, market access offers a more comprehensive reflection of the development level of regional markets and the degree of interconnection between markets [14]. According to the theory of New Economic Geography, market access reshapes the spatial configuration of economic geography and thereby exerts a multi-dimensional impact on the quality of foreign trade [15]. As market access improves, it reduces trade costs and expands firms’ access to external markets and resources. This, in turn, facilitates the cross-regional flow of goods, information, and production factors, thereby creating essential conditions for trade upgrading [16]. On the one hand, enhanced market access offers firms a broader market reach but intensifies competitive pressure, incentivising them to shift toward more advanced and technology-intensive production activities. This transition drives industrial structure advancement, which increases the complexity, innovation content, and value-added of export products—forming a pathway for improving trade quality. On the other hand, the spatial characteristics of market access promote the agglomeration of related industries. Such agglomeration facilitates knowledge spillovers, supplier-buyer matching, and economies of scale, helping firms overcome capability constraints and further enhancing trade efficiency. While both industrial advancement and economic agglomeration serve as key mechanisms through which market access translates into improved trade quality, the effectiveness of this transformation largely depends on the efficiency of resource allocation across sectors [17]. Regions with a higher level of industrial structure rationalization—characterized by more balanced and coordinated distribution of production factors—are better positioned to capitalize on the connectivity advantages brought by improved market access [18]. Rationalization enhances this process by reducing internal inefficiencies and smoothing inter-sectoral factor mobility, thereby conditioning the extent to which market access can generate sustained momentum for foreign trade development. Together, these mechanisms constitute an integrated and interdependent framework.

Early studies have shown that improving transportation infrastructure exerts a cost-reduction effect on foreign trade, whereby the more convenient the transportation, the higher the level of foreign trade. However, further investigations reveal that transportation convenience does not necessarily stimulate export activities, especially at the initial stage of infrastructure improvement. Instead, it often encourages regions and firms to prioritize the development of domestic trade, a phenomenon known as the “domestic trade substitution effect” [13]. Only after the accumulation of network externalities and improvements in factor allocation efficiency does the synergy between domestic and foreign trade gradually emerge, driven by economies of scale. A similar view suggests that transportation infrastructure, through a chain reaction of “cost reduction – market integration – innovation spillovers,” may lead to a nonlinear trajectory in the quality of foreign trade, characterized by an initial decline followed by an eventual rise. The underlying dynamic lies in firms’ strategic adjustments within the process of domestic market integration: during the early stage, the expansion of domestic trade crowds out resources for export, whereas in the later stage, technological spillovers and optimized resource allocation fostered by market integration create new momentum for export upgrading [19]. These perspectives correct the linear perception that transportation infrastructure improvement automatically promotes exports and instead reveal the stage-sensitive nature of its influence—offering a fresh lens for examining the dynamic and nonlinear relationship between market access and foreign trade development, which is the focus of this paper.

### 2.1. The nonlinear relationship between market access and high-quality development of Foreign trade

Existing research has shown theoretical divergences regarding the economic effects of market access, especially in light of its stage-dependent mechanisms. In the initial stage (when market access is relatively low), improvements in transportation infrastructure primarily serve the integration of domestic markets, and the “domestic transportation advantage effect” dominates. During this phase, the marginal returns from domestic trade exceed those from foreign trade, and a substitution relationship exists between the two, which is further reinforced by infrastructure development [20]. Regarding trade scale, the vast size of the domestic market drives firms to allocate more resources to domestic transactions [21]. Furthermore, early improvements in market access may result in “over-connection” with international markets, intensifying resource competition and homogenized competition. Excessive reliance on foreign trade at this stage can lead to a high proportion of low-value-added processing trade, thereby suppressing technological upgrading and causing the so-called “low-end lock-in effect” [22], ultimately hindering urban foreign trade’s high-quality development.

The underlying mechanism undergoes a fundamental shift in the mature stage (when market access is relatively high). The efficient integration of transportation networks with global supply chains enhances the outward orientation of regional economies, while the innovation ecosystems fostered by agglomeration drive exports toward the higher end of the value chain [23]. This dynamic evolution is consistent with the stage-specific characteristics of factor endowment structure upgrading proposed by New Structural Economics. Empirical research also confirms that once market access exceeds a certain threshold, its positive effect on export technological sophistication becomes significantly more pronounced [24]. Based on this, the paper argues that the relationship between market access and the quality of foreign trade exhibits a U-shaped curve characterized by an apparent “middle-zone disadvantage” effect. Accordingly, the following hypothesis is proposed:

**H1:** The impact of market access on the high-quality development of foreign trade exhibits a positive U-shaped pattern with a critical threshold effect. When market access is below the threshold, its influence is dominated by suppression; once market access surpasses the threshold, its promotive effect becomes significantly stronger.

### 2.2. The moderating role of industrial rationalization

Industrial structure rationalization and advancement represent two core dimensions of industrial structure evolution, each characterized by distinct trends and mechanisms [25,26]. Accordingly, this study divides the industrial structure effect into two dimensions for empirical analysis: industrial rationalization and industrial advancement.

According to industrial structure theory, industrial rationalization primarily concerns the coordination among industries and resource utilization efficiency. It refers to the process by which factor resources are gradually reallocated from industries (or enterprises) with lower allocation efficiency to those with higher efficiency [27]. This process reflects the overall improvement of resource allocation efficiency within a region, highlighting the evolution of intensive production, specialized division of labor, and efficient resource configuration in the industrial system [28]. From the perspective of factor endowment theory, improved market access enhances the ease of factor flows and exchanges between cities and diverse markets, providing a practical foundation for resource allocation and factor mobility at the inter-city level. Therefore, cities with a higher degree of industrial rationalization are better positioned to activate the mechanism through which market access facilitates factor flows and reduces trade costs, thereby amplifying the marginal positive effect of market access on foreign trade development.

Conversely, regions with a lower level of industrial rationalization often exhibit fragmented industrial structures, resource misallocation, and weak industrial chain coordination efficiency, leading to suboptimal industrial configurations and a higher likelihood of overcapacity issues [29]. In such regions, improving market access—by enhancing logistics efficiency and external market connectivity—can rapidly open supply-demand channels, promote dual-value chain division of labor domestically and internationally, and compensate for the drawbacks of a poorly rationalized industrial structure, ultimately expanding foreign trade [30]. This mitigates the negative impact of market access on trade quality that stems from the “domestic transportation advantage effect” and the “domestic-foreign trade substitution effect.” Based on this, the following hypothesis is proposed:

**H2:** The higher the degree of industrial rationalization, the stronger the positive impact of market access on the high-quality development of foreign trade.

### 2.3. The industrial advancement effect

Industrial advancement emphasizes the evolution of industries toward higher value-added and higher technology-intensive trajectories. It refers to the gradual advancement of the industrial structure toward a more sophisticated state, aiming for long-term optimization and upgrading the overall economic structure [31]. According to New Structural Economics, market access can facilitate the iterative advancement of the industrial structure, and studies have shown that such advancement can significantly improve the quality of foreign trade [32].

Market access improvements reduce transportation costs and information asymmetry, making it easier for enterprises to enter domestic and international markets [33]. This compels firms to select industries aligned with local comparative advantages and to focus on upgrading those industries, thereby driving the process of industrial structure advancement. At the same time, market access expands the market size, enables industries to achieve economies of scale, lowers unit costs, and facilitates the accumulation of capital and technological capabilities—all of which drive the transition from low-end manufacturing toward technology-intensive industries [34].

The dynamic comparative advantage enhancement and global value chain climbing effects brought by industrial advancement not only increase the export value-added but also optimize the export structure, enabling regions with more advanced industrial structures to receive more trade connections from other areas [35] and promoting the high-quality development of regional foreign trade. Based on this, the following hypothesis is proposed:

**H3:** Market access promotes the high-quality development of foreign trade through the transmission effect of industrial advancement.

### 2.4. The economic agglomeration effect

Market access can also enhance the high-quality development of urban foreign trade by fostering and reinforcing economic agglomeration. According to new economic geography and regional development theory, improvements in market access facilitate transportation, logistics, and information networks, thereby reducing factor mobility and transaction costs. This attracts capital, technology, and labor to concentrate spatially, promoting the clustering of economic activities [36].

First, improvements in market access significantly reduce transaction costs in regions with locational disadvantages, thereby increasing the feasibility of firm entry and operations, and encouraging industries and populations to concentrate in areas with higher accessibility. Stronger economic agglomeration enables firms to achieve economies of scale and scope through sharing production factors, infrastructure utilization, and supply chain collaboration, which in turn improves production efficiency and resource allocation efficiency [37]. Furthermore, agglomeration contributes to the formation of complete industrial chains and supporting service systems, which substantially enhance foreign trade enterprises’ responsiveness to market demand and their production flexibility and customization capacity. This improves the quality and diversification of export products and creates favorable conditions for knowledge spillovers and technological diffusion. The competitive and cooperative dynamics among regional firms further accelerate the dissemination and application of innovations, directly raising foreign trade products’ added value and technological content [38]. Consequently, foreign trade shifts from mere quantitative expansion toward qualitative improvement.

**H4:** Improving market access fosters economic agglomeration, indirectly promoting the high-quality development of foreign trade.

## 3. Empirical design and data sources

### 3.1. Data sources

This study constructs a balanced panel dataset covering 260 prefecture-level cities in China from 2014 to 2023 after excluding county-level cities and cities with excessive data gaps. The data are sourced from multiple authoritative channels, including the *China City Statistical Yearbook*, the EPS database, the CNRDS database, the CEIC China Economic Database, and each province and municipality’s statistical yearbooks.

The fundamental data for calculating market access are mainly derived from OpenStreetMap’s electronic maps of China’s transportation infrastructure network from 2014 to 2023 and the *China City Statistical Yearbook*. Drawing upon the research framework of Donaldson and Hornbeck [39], this study employs the minimum transportation cost of goods between cities as the core indicator to measure the expansion of transportation infrastructure. Combined with the methodology proposed by Baum-Snow et al. [14], the shortest inter-city transportation time is adopted as the core variable for calculating the Market Access (MA) index.

Using China’s road network electronic maps, Geographic Information System (GIS) software tools, including ArcMap 10.8.1 and ArcGIS Pro, were employed to extract the annual traffic routes between any two cities. Based on constructing a road network and an origin-destination (OD) cost matrix, the Dijkstra shortest path algorithm was applied to calculate the optimal driving time between city nodes for each year. Finally, the shortest travel time was integrated with the destination city’s GDP and population data to construct the Market Access index, following the approach detailed below.

### 3.2. Variable measurement

#### 3.2.1. Explained variable.

The high-quality development of foreign trade is a comprehensive concept that any single indicator, such as trade volume, trade structure, or trade efficiency, cannot adequately represent. Academic research has no unified standard for the comprehensive measurement and evaluation of foreign trade quality. Based on the requirements of China’s “14th Five-Year Plan for High-Quality Development of Foreign Trade” and existing research on high-quality trade development indicators [2], this study develops a comprehensive evaluation system consisting of four dimensions: trade development environment, trade development conditions, trade development capacity, and trade cooperation, along with 10 sub-indicators. The system provides a detailed and systematic assessment of the high-quality development of foreign trade in 260 cities in China. (Table 1). The entropy weight method is employed to calculate the high-quality development index of urban foreign trade, thereby reducing the influence of subjective judgment. Due to significant regional differences in trade development among Chinese cities, the trade quality index will show a significant right-skewed distribution. However, this indicates that the level of high-quality trade development reflected by the index is both expected and reasonable. A few cities exhibit outstanding innovation, openness, and cooperation performance, while most remain in early development stages. To alleviate this skewness in regression analysis, we apply a logarithmic transformation to the index, ensuring consistency with the log specification of the market access variable and improving model robustness.

**Table 1:** Evaluation Index System for High-Quality Development of Foreign Trade.

Primary Dimension	Secondary Indicator	Tertiary Indicator	Indicator Description	Indicator Type
Trade Development Environment	Socioeconomic Environment	Per Capita GDP	GDP/ Total Population	Positive
		Economic Volatility	Regional GDP Growth Rate	Positive
		Unemployment	Registered Urban Unemployment Rate	Negative
Trade Development Conditions	Factor Allocation Efficiency	Education Expenditure Ratio	Education Expenditure/ Fiscal Expenditure	Positive
		Innovation Efficiency	Students in Higher Education per 10,000 People	Positive
		Science and Technology Expenditure Ratio	Science & Tech Expenditure/ Fiscal Expenditure	Positive
Trade Development Capacity	Trade Development Scale	Trade Dependence Ratio	Total Trade Volume/ GDP	Positive
		Total Import and Export	Imports + Exports	Positive
Trade Cooperation Level	Partnership	Actual Use of Foreign Direct Investment (FDI)	—	Positive
		Number of New Contracts Signed Annually	—	Positive

**Fig 1:**
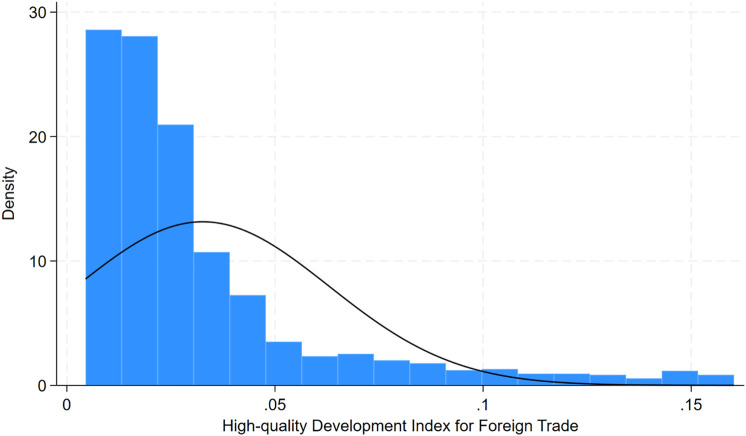
The density of the “High-quality Development of Foreign Trade Index”.

The high-quality trade development index (*Trade*) distribution is right-skewed (Fig 1). This skewness primarily arises from the significant heterogeneity in trade development levels across the 260 cities in China, with a small number of highly developed cities (such as Shenzhen and Shanghai) disproportionately raising the upper tail of the distribution. The fundamental cause of this skewed characteristic lies in the reality of urban development in China. Among the 260 cities, most are medium-sized or small, with only a few megacities (such as Shenzhen, Shanghai, and Guangzhou) highly developed in foreign trade and innovation. This leads to scores being highly concentrated in the middle or lower value range, with only a few cities achieving very high scores, raising the maximum value and causing the distribution to skew to the right. However, this is consistent with Chinese cities’ current foreign trade development.

**(2) Explanatory Variable**

Following the methodology of Donaldson and Hornbeck (2017), the Market Access index for each city-year combination is calculated using the following formula:


$$\begin{array}{c} MarketAccess_{ct}=\sum {\mathrm{\backslash nolimits}}_{c^{\prime }\neq c}\tau_{cc^{\prime }t}^{-\theta }N_{c^{\prime }t} \end{array}$$ (1)

Where *N*_*c’t*_ represents the economic scale (proxied by GDP) of city *c’* in year *t*, this study follows Donaldson and Hornbeck by using the population as an alternative proxy for economic scale in supplemental calculations to test robustness. The parameter *θ* represents trade elasticity, which is set to 8.22 by established studies [39,40]. The variable *τ*_*cc’t*_ represents the iceberg cost—the implicit cost of transporting goods from city *c* to city *c’* in year *t*.

The iceberg cost is computed as follows:


$$\tau_{cc^{\prime }t}=\text{1}+\rho (hours\;of\;travel\;time{)}_{cc^{\prime }t}^{\text{0}.\text{8}}$$ (2)

where $(hours\;of\;travel\;time{)}_{\;cc^{\prime }t}$ denotes the shortest transportation time between cities *c* and *c′* in year t, and *ρ* is a given parameter set at 0.008 following the approach of Baum-Snow et al. [14]. This calculation method leverages detailed road network data to estimate the shortest transportation paths between cities, even without actual firm-level transportation cost records. The functional form also effectively captures the dynamic influence of national road network upgrades on economic indicators. Using an exponent of 0.8 for the minimum transport time ensures the iceberg cost behaves as a concave function, reflecting the positive association between iceberg costs and transportation time and accurately depicting the feature of diminishing marginal transport costs as total travel time increases.

**(3) Moderating Variable**

The level of industrial rationalization is measured using a Theil index-based method [41], which has been widely recognized for its ability to capture the efficiency of resource allocation across industries [42–44]. The industrial rationalization index (*TL*) is calculated as follows:


$$\begin{array}{c} TL_{ct}=\sum {\mathrm{\backslash nolimits}}_{i=\text{1}}^{I}\left(\frac{Y_{cit}}{Y_{ct}}\right)ln\left(\frac{Y_{cit}/L_{cit}}{Y_{ct}/L_{ct}}\right) \end{array}$$ (3)

Where *TL* represents the industrial rationalization index for city *c* in year *t*$Y_{cit}$ is the output of industry *i* in city *c* at time *t*$Y_{ct}$ denotes the to*t*al industrial output of city *c*$L_{cit}$ and $L_{ct}$represent the employment in industry *i* and the total employment across all industries in city *c* at time *t*, respectively. The closer *TL* is to zero, the higher the level of industrial rationalization, indicating an increasingly balanced and efficient allocation of resources among industries.

**(4) Mechanism Variables**

For the measurement of industrial advancement (*Adv*), this study adopts the improved structural similarity coefficient method, also known as the cosine angle method [45]. This convenient method produces intuitive results and supports inter-regional and time-series comparative analysis. The calculation formula is as follows:


$$\begin{array}{c} Adv=\sum {\mathrm{\backslash nolimits}}_{j=\text{1}}^{\text{3}}\sum {\mathrm{\backslash nolimits}}_{i=\text{1}}^{j}\theta_{i}=\sum {\mathrm{\backslash nolimits}}_{j=\text{1}}^{\text{3}}\sum {\mathrm{\backslash nolimits}}_{i=\text{1}}^{j}arccos\left(\frac{X_{\text{0}}\cdot X_{i}}{\left\|X_{\text{0}}\right\|\cdot \left\|X_{i}\right\|}\right),\;i=\text{1},\text{2},\text{3}\; \end{array}$$ (4)

Where $X_{\text{0}\;}$is defined as the three-dimensional vector of the GDP share of value added of primary, secondary, and tertiary industries in a region; $X_{\text{1}}$=(1,0,0), $X_{\text{2}}$= (0,1,0), $X_{\text{3}}$=(0,0,1) represent the theoretical state of the three industries in the state of complete specialization; $\theta_{i}\;$represents the angle between the actual industrial structure vector $X_{\text{0}\;}$and the respective benchmark vectors $X_{i}$; Adv is the index of industrial advancement, and the increase of its value directly reflects the evolution of industrial structure to higher-order form.

In this study, economic agglomeration (*EA*) is measured by GDP per unit of land area. The economic output per unit of area better reflects the degree of agglomeration of local economic activity. Moreover, considering both market size and regional size helps to mitigate measurement bias arising from geographical differences [46]. The formula is as follows:


$$EA_{ct}=\frac{GDP_{ct}}{Land\;Area_{c}}$$ (5)

In this equation, $EA_{ct}$ denotes the level of economic activity in city c during year t; $GDP_{ct}$ represents the gross domestic product of city c in year t; $Land\;Area_{c}$ is the total land area of city c, which remains constant over time. This specification captures the intensity of economic activity per unit of land, thereby reflecting the spatial agglomeration of economic output while controlling for regional size differences.

**(5) Control Variables**

Following established literature, several control variables are introduced to mitigate the influence of confounding factors on foreign trade quality development:

Economic Development Level (*lnGNI*): measured as the logarithm of regional gross national income. Urbanization Level (*Urb*): represented by the logarithm of the city’s total year-end population. Government Intervention (*Gov*): measured as the ratio of general government expenditure to regional GDP. Financial Development (*Fin*): measured as the ratio of the year-end balance of financial institution loans and deposits to regional GDP. Living Standard (*Live*): measured as the number of hospital beds per 100 residents.

### 3.3. Model specification

In order to empirically examine the nonlinear relationship between market access and the high-quality development of foreign trade at the city level, the following econometric model is constructed:


$$\;\;\;\;\;\;\;\;\;\;\;\;\;{lnTrade}_{ct}=\beta_{\text{1}}ln{ma}_{ct}+\beta_{\text{2}}{\left(ln{ma}_{ct}\right)}^{\text{2}}+\Gamma {Controls}_{ct}+\mu_{c}+\delta_{t}+\varepsilon_{ct}$$ (6)

Where ${lnTrade}_{ct}$ represents the high-quality development index of foreign trade for city *c* in year *t;* ${lnma}_{ct}$ represents the market access of ci*t*y *c* in year *t*${{(lnma}_{ct})}^{\text{2}}$ is the squared term of market access, used to capture the nonlinear relationship;${Controls}_{ct}$ represents a series of control variables; $\mu_{c}$denotes the city fixed effect that does not change over *t*ime for city *c*$\delta_{t}$ denotes the fixed effect that controls for time variation; and $\varepsilon_{ct}$ is the random disturbance term. The descriptive statistics for the variables are shown in Table 2.

**Table 2:** Descriptive statistics.

variable name	N	Mean	SD	Min	Max
*lnTrade*	2600	4.309	6.237	0.449	54.45
*lnma*	2600	17.397	1.174	9.890	19.470
*sqrlnma*	2600	302.656	41.043	97.810	379.200
*TL*	2600	0.295	0.210	0.002	1.271
*Adv*	2600	7.361	0.161	6.695	7.754
*AE*	2600	7.347	1.365	1.944	11.996
*lnGNI*	2600	15.118	0.973	12.547	18.354
*Urb*	2600	5.804	0.912	2.325	8.023
*Gov*	2600	0.224	0.134	0.064	1.124
*Live*	2600	0.512	0.175	0.154	1.145
*Fin*	2600	2.842	1.204	0.704	14.248

## 4. Empirical analysis

### 4.1. Benchmark regression

Before conducting hypothesis testing, this paper uses Stata 17 to perform a correlation test among variables. The results show that there is no significant multicollinearity among the variables. Further multicollinearity tests indicate that the average Variance Inflation Factor (Mean VIF = 2.01) is significantly below the critical threshold of 10, confirming that the model does not suffer from multicollinearity issues.

To test Hypothesis H1, this paper incorporates the linear and quadratic terms of the explanatory variable into the regression and includes city and year fixed effects. The regression results are shown in Table 3.

**Table 3:** Benchmark Regression Results.

	lnTrade				
	(1)	(2)	(3)	(4)	(5)
lnma	−16.627^***^ (1.954)	−8.607^***^ (1.371)	−6.690^***^ (0.931)	−20.130^***^ (1.671)	−4.364^***^ (0.716)
sqrlnma	0.563^***^ (0.060)	0.278^***^ (0.042)	0.214^***^ (0.029)	0.649^***^ (0.052)	0.130^***^ (0.022)
lnGNI		3.481^***^ (0.181)		2.917^***^ (0.160)	2.009^***^ (0.224)
Urb		1.122^***^ (0.164)		0.397^***^ (0.150)	3.856^***^ (1.051)
Gov		15.940^***^ (1.876)		13.053^***^ (1.584)	3.151^***^ (0.762)
Fin		−0.110 (0.103)		−0.018 (0.093)	0.148^**^ (0.065)
Live		13.349^***^ (1.031)		13.245^***^ (0.931)	3.070^***^ (0.512)
_cons	123.071^***^ (15.778)	−4.484 (11.595)	55.873^***^ (7.569)	97.466^***^ (12.101)	−17.647^*^ (9.210)
N	2600	2600	2600	2600.000	2600
City	NO	NO	YES	NO	YES
Year	NO	NO	YES	YES	YES
Provence	NO	NO	NO	YES	NO
R^2^	0.184	0.578	0.978	0.798	0.980
U-test	14.766^***^	15.499^***^	15.652^***^	15.513^***^	16.807^***^

**Note:** *p* < 0.1, **p** < 0.05, ***p*** < 0.01; robust standard errors are reported in parentheses.

“U-test” indicates the estimated turning point from the U-shaped test.

First, without fixed effects, both cases without and with control variables are tested to examine the effect of market access on the high-quality development of urban foreign trade. The results are presented in Columns (1) and (2) of Table 3. In both cases, the coefficient of the linear term is significantly positive, while the coefficient of the quadratic term is significantly negative. This provides preliminary evidence that the relationship between market access and the high-quality development of urban foreign trade exhibits a “U-shaped” pattern.

Column (3) includes city-year fixed effects, while columns (4) and (5) include province-year fixed effects and city-year fixed effects after controlling for the control variables. The coefficients of the linear term remain significantly positive, while the coefficients of the quadratic term remain significantly negative. This indicates that Hypothesis H1 is preliminarily supported—namely, the relationship between market access and the high-quality development of foreign trade shows a positive U-shaped pattern.

The estimated coefficient of market access is significantly negative. At the same time, its squared term is significantly positive, indicating a U-shaped relationship between market access and the high-quality development of foreign trade. This suggests that in the early stages, cities with low market access may face trade constraints such as high logistics costs, weak inter-regional connectivity, and limited spillovers, which inhibit trade upgrading. However, once a certain threshold is crossed, further improvements in market access facilitate more efficient resource allocation, enhanced economies of scale, and integration into broader value chains, thereby promoting a shift toward higher-quality trade.

Since the model includes a quadratic term for the explanatory variable, simply relying on the regression coefficients’ significance is insufficient to determine whether the relationship is a “U-shaped” or “inverted U-shaped” curve. Therefore, this paper further adopts the triple conditions proposed by Lind and Mehlum [47] to verify the existence of the U-shaped relationship.

Condition 1: The coefficient of the quadratic term must be significant. For a positive U-shaped relationship, the quadratic term should be significantly positive.

Condition 2: The turning point of the U-shaped curve must lie strictly within the range of the explanatory variable (9.89, 19.47).

Condition 3: The slopes on both sides of the turning point must differ significantly. For a positive U-shaped relationship, the slope to the left of the turning point should be significantly negative, and the right should be significantly positive.

According to the regression results in Column (4) of Table 3, the coefficient of the quadratic term is significantly positive, satisfying Condition 1. The further test results shown in Table 4 indicate that the turning point value of market access is 16.807, which lies within the range of (9.89 and 19.47), thus satisfying Condition 2. The slope to the left of the turning point is −1.796161, and the slope to the right is 0.692509, both significant at the 1% level, satisfying Condition 3. Moreover, the overall U-shaped relationship is statistically significant at 1%.

**Table 4:** U-Shaped Relationship Test.

Extreme point: 16.80692	Overall test of the presence of a U-shape	Lower bound	Upper bound
Interval		9.889639	19.47387
Slope		−1.796161	0.6925096
t-value	3.91	−6.347702	3.905263
P > t	.0000484	1.31e-10	0.0000484

Combining the regression results and the U-shaped test (Table 4), the impact of market access on the high-quality development of urban foreign trade is indeed a positive U-shaped relationship with a critical threshold effect. Hypothesis 1 is supported.

**Fig 2:**
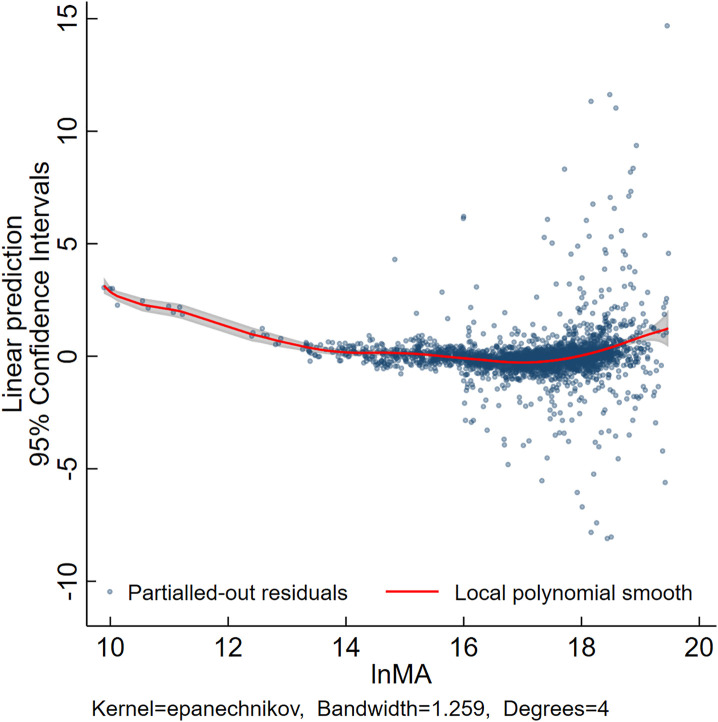
Semiparametric regression with fixed effects.

Building upon the regression analysis, this study constructed a semiparametric regression plot incorporating fixed effects (Fig 2). Using xtsemipar to perform nonparametric fitting on *ln(MA)* while keeping the control variables and fixed effects parametric allows for a more accurate representation of the actual shape relationship between *ln(MA)* and *lnTrade*, without being influenced by pre-specified linear or quadratic model forms.

### 4.2. Endogeneity treatment

As discussed in Section 3, the measurement approach for market access has already minimized the risk of endogeneity to the greatest extent possible. However, to address potential concerns about omitted variable bias and the possibility of reverse causality between market access and the high-quality development of foreign trade, this paper adopts the instrumental variable (IV) method.

Specifically, the lagged values of the explanatory variable and its squared term are used as instruments to perform two-stage least squares (2SLS) regression to test for the presence of reverse causality [48]. This is one of the most commonly used methods for addressing endogeneity and also avoids the issue of “forbidden regression” in nonlinear models when using a single instrument [49].

In addition, to further strengthen identification and test robustness, this paper constructs another instrumental variable (*IV3*): the average market access level of other cities within the same province, excluding the target city itself and its directly adjacent cities. This instrument satisfies three critical conditions. First, relevance: cities within the same province often share transportation networks, policy environments, and economic planning frameworks, which jointly influence their accessibility. Second, exogeneity: by excluding the target city and its neighbors, the instrument avoids capturing local shocks or spatial spillovers that may directly influence foreign trade. Third, exclusion restriction: the instrument only affects the dependent variable (foreign trade quality) through its influence on the market access of the target city, rather than through any direct channel.

Table 5 shows the regression results. After applying the instrumental variable approach, the second-stage regression results show that the coefficient of the linear term for market access remains significantly positive at the 1% level. In comparison, the coefficient of the quadratic term remains significantly negative at the 1% level. This confirms that the positive U-shaped relationship still holds.

**Table 5:** Endogeneity Treatment Regression Results.

	(1)		(2)		(3)	
	First stage	Second stage	First stage	Second stage	First stage	Second stage
	lnma	lnTrade	lnma	lnTrade	lnma	lnTrade
L.lnma	1.023^***^ (0.097)					
L.sqrlnma	−0.010^**^ (0.003)					
L2.lnma			0.855^***^ (0.136)			
L2.sqrlnma			−0.015^***^ (0.004)			
IV3					5.607^**^ (2.497)	
sqrIV3					−0.467^***^ (0.088)	
lnma		−4.968^***^ (1.222)		−4.931^***^ (1.408)		−3.211^**^ (1.318)
sqrlnma		0.143^***^ (0.038)		0.128^***^ (0.042)		0.119^***^ (0.042)
Controls	YES	YES	YES	YES	YES	YES
City	YES	YES	YES	YES	YES	YES
Year	YES	YES	YES	YES	YES	YES
Kleibergen-Paap rk LM	–	1321.866^***^	–	486.322^***^	–	179.362^***^
Kleibergen-Paap rk Wald F	–	1340.518	–	232.796	–	86.101

**Note:** *p* < 0.1, **p** < 0.05, ***p*** < 0.01; robust standard errors are reported in parentheses.

“U-test” indicates the estimated turning point from the U-shaped test.

### 4.3. Robustness tests

#### 4.3.1. Measurement of market access.

Referring to Donaldson and Hornbeck [39], this study uses the population size of destination cities to replace GDP as the economic scale indicator for recalculating the market access index. As shown in Column (1) of Table 6, the regression results remain robust after substituting the measurement method for market access.

**Table 6:** Robustness Tests.

	(1)	(2)	(3)	(4)
	lnTrade	lnTrade	lnTrade	lnTrade
lnma		−5.214^***^ (1.184)	−4.668 (4.394)	−4.266^***^ (0.374)
sqrlnma		0.160^***^ (0.037)	0.445 (0.294)	0.114^***^ (0.011)
lnma_2	−2.653^***^ (0.949)			
sqrlnma_2	0.077^***^ (0.033)			
lnma3			−0.012 (0.008)	
Controls	YES	YES	YES	YES
City	YES	YES	YES	YES
Year	YES	YES	YES	YES
$R^{\text{2}}$	0.979	0.983	0.980	–
U-test	16.456^**^	16..283^***^	–	16.636^***^

**Note:** *p* < 0.1, **p** < 0.05, ***p*** < 0.01; robust standard errors are reported in parentheses.

“U-test” indicates the estimated turning point from the U-shaped test.

#### 4.3.2. Winsorization of samples.

To reduce the influence of outliers on the estimation results, the main variables are winsorized at the 1% level on both ends. As shown in Column (2) of Table 6, after winsorization, the direction and significance of the coefficients for the linear and quadratic terms of market access remain unchanged, confirming the robustness of the findings.

#### 4.3.3. Inclusion of the cubic term.

According to the research of Haans et al [50], if the cubic term of an explanatory variable is significant after being included in the regression, the relationship between the independent and dependent variables would follow an S-shaped curve rather than a U-shaped curve. After adding the cubic term of market access (*lnma3*), the regression results are shown in Column (3) of Table 6. The coefficient of the cubic term is not significant, indicating that the relationship is not S-shaped, and the positive U-shaped relationship remains robust.

#### 4.3.4. Double machine learning (DDML).

Since the baseline model is nonlinear, traditional econometric methods may suffer from model misspecification. The double machine learning (DDML) approach proposed by Chernozhukov [51] effectively captures nonlinear relationships between variables. It offers significant advantages in handling nonlinear data, reducing the risk of model misspecification, and improving estimation reliability. Therefore, this study re-estimates the model using a random forest algorithm. As shown in Column (4) of Table 6, the signs and significance levels of the coefficients for both the linear and quadratic terms of market access remain unchanged, confirming the robustness of the results.

### 4.4. Heterogeneity analysis

The heterogeneous relationship between market access and the high-quality development of foreign trade across different regions and city types reflects geographical disparities and more profound structural and institutional differences among urban clusters. As shown in Table 7, the U-shaped relationship is more pronounced and statistically significant in central, western, and inland cities. In contrast, it appears weaker or insignificant in eastern and coastal/border regions. This divergence suggests that the marginal effects of improved market access are stage-dependent and are significantly influenced by initial access levels and the maturity of industrial development.

**Table 7:** Heterogeneity Analysis.

	(1)	(2)	(3)	(4)	(5)
	East cities	Central cities	West cities	Coastal and Border Cities	Landlocked cities
	lnTrade	lnTrade	lnTrade	lnTrade	lnTrade
lnma	−1.360 (2.069)	−3.316^**^ (1.610)	−2.933^***^ (0.487)	−1.929 (3.431)	−5.148^***^ (1.301)
sqrlnma	0.047 (0.064)	0.088^*^ (0.049)	0.053^***^ (0.015)	0.041 (0.103)	0.144^***^ (0.039)
Controls	YES	YES	YES	YES	Controls
N	1140	710	750	1530	1090
City	YES	YES	YES	YES	YES
Year	YES	YES	YES	YES	YES
$R^{\text{2}}$	0.980	0.950	0.958	0.981	0.980

**Note:** *p* < 0.1, **p** < 0.05, ***p*** < 0.01; robust standard errors are reported in parentheses.

Specifically, central and western regions, characterized by historically insufficient infrastructure investment and lower levels of regional connectivity, generally exhibit a lower degree of market integration. In these areas, improvements in market access can effectively reduce trade barriers, unleash the potential for cross-regional factor flows, and promote export expansion. Moreover, since the industrial systems in these regions are still in a developmental phase, there is considerable room for structural transformation. Enhanced accessibility thus facilitates industrial upgrading toward higher value-added sectors. Consequently, the U-shaped impact of market access on trade quality observed in these regions reflects a “latecomer advantage”. It aligns with the theoretical predictions of new structural economics regarding the co-evolution of accessibility and industrial upgrading.

By contrast, cities in the eastern and coastal regions already have well-developed infrastructure, high connectivity, and a high degree of openness. In such “mature” economies, the marginal returns to further improvements in market access tend to diminish. At the same time, these cities may face issues such as excessive agglomeration, environmental constraints, or institutional rigidity, which weaken the responsiveness of trade quality to enhanced access. In some cases, trade saturation and intensified competition may even offset the potential benefits of improved connectivity—particularly in cities where export structures are already optimized and deeply embedded in global value chains.

Similarly, inland cities, long constrained by limited access to external markets and transportation corridors, start from a lower baseline of accessibility. As a result, they stand to benefit more significantly from improvements in market access. Better connectivity helps alleviate logistical bottlenecks and enables these cities to integrate more easily into domestic and global industrial chains, thereby enhancing the feasibility of trade upgrading. These spatial differences underscore the crucial role of market access in narrowing regional development gaps and fostering inclusive growth in foreign trade.

## 5. Mechanism testing and moderating effect analysis

The mechanism testing method for nonlinear models differs from that of linear models. This paper employs two models (Table 8) for the mechanism analysis.

**Table 8:** Mechanism Effect Testing Models for Quadratic Curves.

Effect Type	Mediating Effect in Quadratic Curves	
Model	Model 1	Model 2
Model Expression	$M=\beta_{\text{0}}+\beta_{\text{1}}X+\beta_{\text{2}}X^{\text{2}}$ $Y=\beta_{\text{3}}+\beta_{\text{2}}X+\beta_{\text{5}}X^{\text{2}}+\beta_{\text{6}}M$ $\text{IND}=\beta_{\text{2}}\times \beta_{\text{6}}$	$M=\beta_{\text{0}}+\beta_{\text{1}}X$ $Y=\beta_{\text{2}}+\beta_{\text{3}}X+\beta_{\text{4}}M+\beta_{\text{5}}M^{\text{2}}$ $\text{IND}=\beta_{\text{1}}\times \beta_{\text{5}}$
Relationship Pattern	Positive U-shaped indirect effect (IND > 0); Negative U-shaped indirect effect (IND < 0).	Positive U-shaped indirect effect (IND > 0); Negative U-shaped indirect effect (IND < 0).
Key Testing Criterion	Bootstrap method, parameter IND is significant.	Bootstrap method, parameter IND is significant.

### 5.1. Industrial advancement effect

The mechanism of industrial structure advancement was tested using Model 1 in Table 9, and the results are reported in Columns (1) and (2) of Table 9. As shown in Table 9, when regressing industrial structure advancement (*Adv*) on market access, both the linear and quadratic terms of market access are significant, with the quadratic term being significantly positive. When foreign trade quality (*lnTrade*) is taken as the dependent variable and Adv is included in the estimation, the coefficient of Adv is significantly positive, while the signs and significance of the coefficients of the linear and quadratic terms of market access remain unchanged. Based on Model 1, this indicates that the effect of market access on the high-quality development of foreign trade operates through the transmission mechanism of economic agglomeration, with industrial agglomeration exerting a positive U-shaped indirect effect.

**Table 9:** Mechanism Testing Results.

	Model 1		Model 2	
	(1)	(2)	(3)	(4)
	Adv	lnTrade	EA	lnTrade
lnma	−0.853^***^ (0.219)	−4.518^***^ (0.712)	0.039^***^ (0.010)	−0.168^**^ (0.085)
sqrlnma	0.025^***^ (0.007)	0.133^***^ (0.022)		
Adv		0.286^**^ (0.106)		
EA				−5.387^***^ (1.538)
sqrEA				0.403^**^ (0.052)
Controls	YES	YES	YES	YES
N	2600	2600	2600	2600
City	YES	YES	YES	YES
Year	YES	YES	YES	YES
$R^{\text{2}}$	0.840	0.978	0.895	0.977

**Note:** *p* < 0.1, **p** < 0.05, ***p*** < 0.01; robust standard errors are reported in parentheses.

### 5.2. Economic agglomeration effect

The mechanism of economic agglomeration was further tested using Model 2 in Table 8, with results presented in Columns (3) and (4) of Table 9. The results in Table 9 show that market access has a significantly positive effect on economic agglomeration (*EA*). When both the linear and quadratic terms of *EA* are included in the model, the quadratic term is significantly positive at the 5% level. Based on Model 2, it can be inferred that industrial structure effects exert a positive U-shaped indirect effect in the relationship between market access and the high-quality development of foreign trade, suggesting that market access influences trade quality through industrial structure advancement.

To further validate the robustness of the mechanisms, the Bootstrap method was employed for testing industrial effects (IND). A total of 1,500 repeated resampling simulations were conducted with a 95% confidence interval. The results, reported in Table 10, show that the indirect effects of both industrial structure and economic agglomeration are significantly positive at the 1% level, and the 95% confidence intervals do not include zero. Therefore, hypotheses 3 and 4 are supported.

**Table 10:** Bootstrap Test Results for Mechanism Models.

Mechanism Variable	Effect Type	Coefficient	z-value	p-value	Upper 95% CI	Lower 95% CI
Adv	Ind_eff	0.290	2.81	0.005	0.1169048	0.5194633
EA	Ind_eff	0.016	3.01	0.003	0.0053928	0.0257255

### 5.3. The moderating effect of industrial rationalization

Based on the benchmark model, this paper introduces interaction terms between market access and industrial rationalization (*TL*) to analyze the moderating effect of industrial rationalization. The estimation results are shown in Table 11. The fitting curve is presented in Fig 1.

**Table 11:** Regression Results of the Moderating Effect.

	(1) lnTrade
lnma	−5.318^***^ (0.866)
sqrlnma	0.1616^***^ (0.027)
TL	−18.562^*^ (8.574)
lnma×TL	2.500^**^ (3.066)
sqrlnma×TL	−0.081^**^ (0.030)
Controls	YES
N	2600
City	YES
Year	YES
$\;R^{\text{2}}$	0.980

**Note:** *p* < 0.1, **p** < 0.05, ***p*** < 0.01; robust standard errors are reported in parentheses.

Since the industrial rationalization index (*TL*) decreases as the level of rationalization increases, it is a negative indicator. The regression results in Table 11 show that the leading effect coefficient of *TL* is significantly negative at the 5% level, indicating that improvements in industrial rationalization can enhance the quality of foreign trade development, which manifests as a rightward and upward shift in the U-shaped curve.

Looking at the interaction term between the linear term of market access and industrial rationalization, the coefficient for the linear term is −5.318 and significant at the 1% level, while the coefficient for the interaction term is 2.500 and significant at the 5% level. This suggests that the more significant the *TL* value (i.e., the lower the level of rationalization), the lower the negative effect of the linear term (the total effect of the linear term = −5.138 + 2.500 × *TL*). This indicates that before the turning point, an improvement in industrial rationalization (*TL*↓) weakened the nonlinear negative effect of market access on the high-quality development of urban foreign trade.

For the interaction term between the quadratic term of market access and industrial rationalization, the coefficient of the quadratic term is 0.161, significant at the 1% level, and the interaction term’s coefficient is −0.081, significant at the 5% level. This shows that the smaller the *TL* value (i.e., the higher the rationalization level), the stronger the positive effect of the quadratic term (the total effect of the quadratic term = 0.161 − 0.081 × *TL*). This implies that beyond the turning point, an improvement in industrial rationalization (*TL*↓) enhances the positive nonlinear effect of market access on the high-quality development of urban foreign trade.

**Fig 3:**
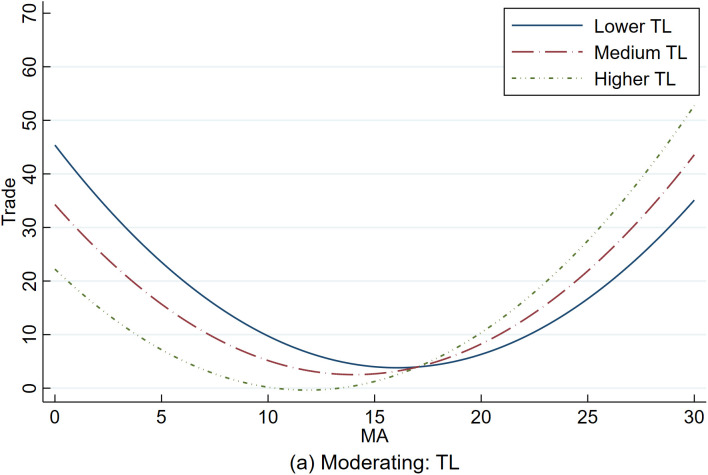
Moderating effect diagram of TL.

Combined with Fig 3, when the level of industrial rationalization is relatively high (*TL*↓), market access improvement after crossing the turning point can, through industrial structure effects and economic agglomeration effects, release tremendous trade potential over the long term and strengthen the positive impact of market access on the high-quality development of urban foreign trade. Conversely, when the level of industrial rationalization is low (*TL*↑), the secondary effect diminishes, the U-shaped curve flattens, and the nonlinear effect of market access on the high-quality development of urban foreign trade is weakened. Therefore, Hypothesis 2 is supported.

## 6. Conclusion and implications

This study empirically investigates the impact of market access on the high-quality development of foreign trade using panel data from 260 Chinese cities from 2014 to 2023. The main findings reveal a significant positive U-shaped relationship between market access and trade quality. In the initial stages, improvements in market access primarily facilitate domestic market integration, leading to a substitution effect where domestic trade crowds out resources for foreign trade, thereby temporarily inhibiting the upgrading of trade quality. However, once market access surpasses a critical threshold (lnma = 16.807), its positive effects dominate. Enhanced connectivity fosters industrial structure advancement, promotes economic agglomeration, and enables regions to integrate into global value chains more effectively, ultimately driving a shift towards high-quality foreign trade development.

Furthermore, mechanism analyses confirm that industrial structure advancement and economic agglomeration are two pivotal transmission channels. Industrial rationalization also plays a positive moderating role. Additionally, heterogeneity analysis reveals that market access in the western and inland regions starts relatively low, but shows strong catching-up potential; as network effects accumulate, its marginal returns become more prominent. This result is consistent with the theoretical inference of dynamic adaptation of factor endowments over time in new structural economics, and it also reveals that the role of market access in promoting trade quality varies significantly at different stages of development.

### 6.1. Implications

Building on the empirical findings, this study advances several policy recommendations to promote the high-quality development of foreign trade:

(1) Differentiated and region-specific infrastructure strategies. Policymakers should adopt a place-based approach tailored to regional development stages. In central, western, and inland cities—currently located on the left side of the U-shaped curve—priority should be given to large-scale and targeted investments in transportation and digital infrastructure to overcome geographical disadvantages and activate the positive cycle of market access. In contrast, for eastern and coastal cities that are likely already beyond the threshold, policy should shift toward optimizing existing networks, upgrading digital connectivity, and improving logistics efficiency to maximize quality gains from their advanced access conditions.
(2) Fostering industrial upgrading and agglomeration in lagging regions. Industrial policies in central and western areas should exploit their strong potential for structural transformation. Governments can guide resource allocation toward high-value-added sectors, establish specialized industrial zones to capture agglomeration economies, and promote technology transfer. These measures can help such regions avoid the “low-end lock-in” trap and accelerate their progression along the U-shaped trajectory.
(3) Enhancing industrial rationalization. Reducing resource misallocation and improving factor mobility efficiency are essential across all regions. A rationalized industrial structure amplifies the benefits of improved accessibility by ensuring that connectivity translates into productive capacity and export upgrading, particularly in regions experiencing rapid improvements in market access.
(4) Promoting coordinated regional development. The results highlight the necessity of narrowing disparities in market access across regions. Policies should encourage collaboration between eastern and western areas through paired assistance, industrial chain relocation, and cross-regional cooperation platforms. Such strategies can help less-developed regions overcome the initial inhibitory phase of market access and achieve leapfrog improvements in trade quality.
